# Striking Similarities Between *Botrytis cinerea* From Non-agricultural and From Agricultural Habitats

**DOI:** 10.3389/fpls.2018.01820

**Published:** 2018-12-05

**Authors:** Marc Bardin, Christel Leyronas, Claire Troulet, Cindy E. Morris

**Affiliations:** Pathologie Végétale, INRA, Montfavet, France

**Keywords:** microbial ecology, epidemiology, gray mold, polyphagous, diversity, aggressiveness, fitness, selection

## Abstract

Investigations into life history of microorganisms that cause plant diseases have been limited mostly to contexts where they are in interaction with plants, and with cropped or otherwise managed vegetation. Therefore, knowledge about the diversity of plant pathogens, about potential reservoirs of inoculum and about the processes that contribute to their survival and adaptation is limited to these contexts. The agro-centric perspective of plant pathogen life histories is incoherent with respect to the capacity of many of them to persist as saprophytes on various substrates. In this context we have investigated the ubiquity of the broad host range necrotrophic fungus *Botrytis cinerea*, outside of agricultural settings and have determined if the populations in these natural habitats can be distinguished phenotypically and phylogenetically from populations isolated from diseased crops. Over a period of 5 years, we isolated *B. cinerea* from 235 samples of various substrates collected in France including rainfall, snowpack, river, and lake water, epilithic biofilms in mountain streams, leaf litter and plant debris, rock surfaces, bird feathers and healthy wild plants from outside of agricultural fields. All substrates except rock surfaces harbored *B. cinerea* leading us to establish a collection of purified strains that were compared to *B. cinerea* from diseased tomato, grapes and various other crops in France. Phylogenetic comparisons of 321 strains from crop plants and 100 strains from environmental substrates based on sequences of 9 microsatellite markers revealed that strains from crops and the environment could not be distinguished. Furthermore, the genetic diversity of strains outside of agriculture was just as broad as within agriculture. In tests to determine the aggressiveness of strains on tomato stems, the mean disease severity caused by strains from environmental substrates was statistically identical to the severity of disease caused by strains from tomato, but was significantly greater than the severity caused by strains from grape or other crops. Our results suggest that highly diverse populations of this plant pathogen persist outside of agriculture in association with substrates other than plants and that this part of their life history is compatible with its capacity to maintain its potential as plant pathogen.

## Introduction

Investigations into the life history of microorganisms that can cause disease to plants have been limited mostly to contexts where they are in interaction with plants, and even more frequently with cropped or otherwise managed vegetation (Morris et al., 2009). Therefore, knowledge about the diversity of plant pathogens, about potential reservoirs of inoculum and about the processes that contribute to survival and adaptation of plant pathogens is limited to these contexts. By contrast, there are many examples of human pathogens for which habitats and biological activities are known outside the strictly medical context (Morris et al., 2009). The agro-centric perspective of plant pathogen life histories is incoherent with respect to the capacity of many plant pathogens to persist as saprophytes on various substrates. Thus, various plant pathogens, including bacteria, fungi and viruses have been detected in non-agricultural habitats or in the absence of host plants (Morris et al., 2009). For instance, recently the fungal species *Fusarium oxysporum*, a major fungal plant pathogen, has been isolated in tap water (Edel-Hermann et al., 2016). However, apart from the bacterial species *Pseudomonas syringae* (Morris et al., 2013), and the oomycete genera *Phytophthora* (Hansen et al., 2012), the ecology and evolutionary history of plant pathogens at spatiotemporal scales wider than their strict interaction with plants (either cultivated or wild) has not been extensively studied.

The aerially disseminated fungus *Botrytis cinerea* is considered to be a broad-host range necrotrophic pathogen able to attack about 586 plant genera, mostly dicots (Elad et al., 2016). It is also capable of growing as a saprophyte on dead and decaying plant matter (Williamson et al., 2007) and it probably has an underestimated facultative endophytic behavior, i.e., it survives in host plant tissue without generating disease symptoms (Van Kan et al., 2014). Additionally this fungus has been detected in non-agricultural contexts such as outdoor or indoor air in agricultural or urban settings (Bartlett et al., 2004; Lugauskas and Krikstaponis, 2004; Leyronas and Nicot, 2013), in clouds (Amato et al., 2007), in precipitation (Monteil et al., 2014), in agricultural or non-agricultural soils (Azmi and Seppelt, 1998; Leyronas et al., 2015c), on rocks and monuments (Gaylarde and Morton, 1999), in stocks of hydrocarbons (Gaylarde et al., 1999), on external or in internal parts of insects (Fermaud and Lemenn, 1989; Louis et al., 1996), on pelage of small mammals (Shchipanov et al., 2006) and human hairs (Ali-Shtayeh et al., 2001). However, to our knowledge, the genetic diversity and the pathogenic potential of saprophytic *B. cinerea* strains isolated from non-agricultural habitats have never been studied.

Recent research is revealing that natural environments harbor strains of microorganisms that have likely given rise to strains that have emerged in plant disease epidemics (Hansen et al., 2012; Monteil et al., 2016). In this context, we have assessed the ubiquity of the fungus *B. cinerea* outside of agricultural settings and have determined if the populations in these natural habitats can be distinguished phenotypically and phylogenetically from populations isolated from diseased crops. The objectives of the present study were (i) to determine the presence and the abundance of *B. cinerea* in various non-agricultural habitats, (ii) to evaluate the genetic diversity and the phenotypic diversity of the strains collected, and (iii) to compare this diversity with strains collected from crops.

## Materials and Methods

### Isolation of *B. cinerea* From Non-agricultural Habitats

Over a period of 5 years (2005–2010), we collected 235 samples of various substrates, including rainfall, snowpack, fresh water from rivers and lakes, epilithic biofilms in mountain streams, rocks, leaf litter and plant debris, healthy wild plants from outside of agricultural fields and feathers from the great tit (*Parus major*) (Table 1). Most of samples originated from the Provence-Alpes-Côte d’Azur region in France. Some of the strains obtained from rainfall and snowfall were part of a previous study (Monteil et al., 2014).

**Table 1:** Quantification of the number of *Botrytis cinerea* strains collected in non-agricultural habitats (environmental strains) and number of strains tested for different traits.

		Number of			Number of strains
	Number of	samples	Number of *B. cinerea*	Number of	tested for their	Number of strains
	samples	containing	strains detected	genotyped	aggressiveness	tested for other
Origin of samples	collected	*B. cinerea*	on medium	strains	on tomato	phenotypic traits
Precipitation	35	18	84	69	84	11
Snowpack	35	7	13	10	13	5
Fresh water	56	14	18	12	11	3
Epilithic biofilm	18	3	3	2	0	0
Litter	33	4	4	3	1	1
Rock surfaces	6	0	0	0	0	0
Plant debris	9	1	1	1	1	1
Asymptomatic wild plants	37	6	10	7	10	2
Bird feather	6	3	8	5	8	1
Total	235	56	141	109	128	24


Each sample was plated on Petri plates containing the semi-selective *Botrytis* Spore Trap Medium (BSTM) (Edwards and Seddon, 2001). The Petri plates were sealed with parafilm and incubated at 20°C in daylight for 14 days to allow the development of fungal colonies. The colonies with mycelium resembling that of *B. cinerea* were individually sub-cultured on Potato Dextrose Agar (PDA) and their identity was confirmed 7 days after transplanting by observing characteristic asexual sporulation of *B. cinerea* (Barnett, 1998). Dry sterile cotton swabs were rubbed on sporulating plates to collect *B. cinerea* spores. The swabs were stored at -20°C until isolate purification.

All isolates were purified and single-spored in a classical way (Leyronas et al., 2012) prior to their genotypic and phenotypic characterization. Hereafter these characterized single spore isolates will be referred to as “environmental strains.”

### Strain Genotyping

A subset of 109 strains collected from non-agricultural habitats were genotyped (Table 1). They were compared to 327 agricultural strains sampled from lettuce and tomato plants grown in several greenhouses in the South of France (Leyronas et al., 2015a,b,c).

Genomic DNA was extracted from aliquots of 15 mg lyophilized fungal material (harvested from two-week old cultures on Potato Dextrose Agar), following the DNeasy Plant extraction Kit protocole (Qiagen). The nine microsatellite markers designed for *B. cinerea* by Fournier et al. (2002) were amplified following the protocol described by Leyronas et al. (2015b). To determine the size of the microsatellites, the PCR products were scanned with the help of an ABI 3730 sequencer (Applied Biosystems). GeneMapper software version 4.1 (Applied Biosystems) was then used for the microsatellite size analysis. Complete microsatellite size profiles (referred to as “haplotypes” hereafter) were obtained for 109 environmental strains and 327 agricultural strains.

### Genetic Characteristics of Strains

In a first step, the strains with the private allele at microsatellite locus BC6 associated with the cryptic species *B. pseudocinerea* (Walker et al., 2011) were removed from further analyses. This private allele is found only in *B. pseudocinerea* strains and never in *B. cinerea* strains. Then, in order to compare the *B. cinerea* strains sampled from the different reservoirs, several indices of genetic diversity were used. The software FSTAT version 2.9.3 (Goudet, 1995) was used to compute allelic richness (corrected for the smallest sample size) and unbiased gene diversity per locus. Unbiased gene diversity (Hnb) and allelic richness (average over the nine loci) were computed separately for the strains collected in the different reservoirs with the Genetix software (Belkhir et al., 1996–2004). The number of different multilocus haplotypes (MLH) was computed with GenClone 1.0 software (Arnaud-Haond and Belkhir, 2007). We used the index of haplotypic diversity (based on the number of individuals and the number of distinct MLH), which estimates the proportion of haplotypes present in a population and takes a value of 1 when a population is composed exclusively of unique haplotypes (Arnaud-Haond et al., 2007).

### Phylogenetic Relationships Between Strains From Non-agricultural Habitats and From Crops

To assess the relationships between the different strains, we computed a neighbor-joining (NJ) tree with the program POPULATIONS (version 1.2.32 provided by Olivier Langella, Quantitative Genetics and Evolution, Gif-sur-Yvette, France). The NJ-tree was based on the distances of Cavalli-Sforza and Edwards (1967), computed from our microsatellite loci. The tree was visualized and edited with TREEVIEW (Page, 1996). All analyses were conducted on data sets excluding clone replicates.

In addition, Arlequin version 3.5 (Excoffier et al., 2005) was used to assess genetic differentiation between strains collected from non-agricultural habitats (NH) and from crops by computing RST values as suggested by Slatkin (1995) for microsatellite data. The clonally corrected data set was used.

### Aggressiveness of Strains on Tomato Plants

The aggressiveness of a subset of 128 environmental strains (Table 1) was assessed on 8-week old tomato plants cv. Monalbo (INRA). Tomato plants were grown in a greenhouse and watered daily with a nutrient solution as described previously (Decognet et al., 2009). Each strain was inoculated on three plants. On each plant, three leaves were removed, leaving 1 cm petiole stubs on the stems and the wounds were inoculated with 10 μL aliquots of spore suspension. The spore suspensions were prepared from two-week old cultures on PDA and were adjusted to 10^6^ spores mL^-1^. All plants were incubated in a growth chamber with a photoperiod of 14 h, with a light intensity of 162 μmol m^-2^ s^-1^, maintained at 21°C with a relative humidity above 90%. The length of resulting stem lesions was monitored daily from the 3rd to the 7th day after inoculation and these data were used to compute the area under the disease progress curves (AUDPC). Due to a limited place in the growth chamber, strains were tested in series of 10–15 strains, and in each series, strain BC1 was used as a reference to calculate an index of aggressiveness (IA) for each strain, relative to that of this strain (Decognet et al., 2009), as follows:

$$IA=100^{*}\left({AUDPC}_{strain}/{AUDPC}_{BC1}\right)$$

with AUDPC_strain_ being the value of AUDPC computed for the tested strain and AUDPC_BC1_ the value of AUDPC obtained for the reference strain BC1. Two to three independent repetitions of the test were realized for each strain.

The aggressiveness of environmental strains was compared to that of 156 single-spored agricultural strains having a wide diversity regarding year, region of isolation and host plants. All these strains were collected from 1988 to 2008 with a majority collected after 2000 (108 strains). Regarding their geographical origin, 149 strains were sampled from France, of which 122 were from the southern part of the country. In addition, four strains came from Italy, two from Syria and one from Portugal. They were collected from various diseased plants: 101 from tomato, 33 from grape, and 22 from other plants including rose, cucumber, strawberry, artichoke, pepper, carrot, onion, asparagus, peach fruit, cherry fruit, kiwifruit, hydrangea, gerbera, cyclamen, and poinsettia.

### Traits of *B. cinerea in vitro*

Mycelial growth, sporulation and sclerotia production were evaluated for a sub-sample of 31 agricultural strains and 24 environmental strains of *B. cinerea* (Table 1), which represent the full range of aggressiveness of the tested strains. Mycelial growth was evaluated by inoculating PDA with a 5 mm-diameter mycelial plug of each *B. cinerea* strain and incubating in a growth chamber (21°C, photoperiod 14 h, 114 μmol m^-2^ s^-1^). Two perpendicular measurements of the diameter of the mycelial colony were performed every day for 3 days and the rate of mycelial growth between the first and the third day of incubation was calculated for each strain. Three plates were inoculated for each strain and the whole experiment was repeated two times. The spore production was determined 14 days after inoculation on PDA medium (21°C, photoperiod 14 h, 114 μmol m^-2^ s^-1^). Spores were scrapped from the media, suspended in water and spore concentration was determined using a hemacytometer. The number of sclerotia produced by each strain of *B. cinerea* on PDA was recorded after five weeks of incubation at 21°C (1 week with a photoperiod of 14 h at 114 μmol m^-2^ s^-1^ and 4 weeks in the dark) and 5 weeks of incubation at 4°C in the dark. For the sporulation and sclerotia production experiments, three replicates were realized for each strain.

### Statistical Analyses

Statistical analyses were performed with Statistica (version 12, Statsoft). Non-parametric tests (Mann and Whitney) were used to determine significant differences between gene diversity and allelic richness of environmental and agricultural strains. To compare the different phenotypic traits evaluated, analysis of variance (ANOVA) were performed. In the case of a significant effect of the tested factor, a comparison of mean was realized using the Newman–Keuls test. The relationship between the various phenotypic traits tested (mycelial growth, sporulation, sclerotia production) and aggressiveness was realized using linear regressions. Statistical inferences were made at the 5% level of significance, unless indicated otherwise.

## Results

### *B. cinerea* Is Ubiquitous in Non-agricultural Habitats

*Botrytis cinerea* was recovered in 56 samples out of the 235 samples collected (Table 1). From these 56 samples, we were able to purify 141 strains of *B. cinerea*. All substrates except rock surfaces harbored *B. cinerea*. The fungus was detected at a rate of 51% in precipitation, 50% from feathers of Great Tits, 21% in fresh water and 20% in snowpack. Detection rates were between 11 and 17% in the other substrates including epilithic biofilm, litter, plant debris and asymptomatic plants.

### Environmental and Agricultural Strains Cannot Be Significantly Differentiated Based on Their Genetic Diversity

Among the 109 genotyped environmental strains and 327 genotyped agricultural strains, 9 and 6 strains, respectively, carried the private allele at microsatellite locus BC6 associated with the cryptic species *B. pseudocinerea* (Walker et al., 2011) and were removed from further analyses. We thus compared the indices of genetic diversity based on the haplotypes obtained for the 100 and 321 remaining strains considered to be *B. cinerea* (Table 2). There was no significant difference between the number of alleles in each microsatellite locus and the gene diversity per locus of environmental strains and agricultural strains (respectively, P_Mann and Whitney_ = 0.60, P_Mann and Whitney_ = 0.79) (Table 3). The global gene diversity and the mean number of alleles per locus were in the same range for both population of strains (Table 2). However, environmental strains had higher haplotypic diversity than agricultural strains (Table 2). Two hundred and seventy different multilocus haplotypes (MLH) were found among the 423 strains analyzed but none of these was shared by environmental strains and agricultural strains.

**Table 2:** Indices of genetic diversity among strains collected from non-agricultural habitats (environmental strains) and from crops (agricultural strains).

	Number of	Number of	Number of
	strains	*B. pseudocinerea*	*B. cinerea*	Gene diversity	Mean number of	Number of	Haplotypic
Origin of strains	genotyped	strains	strains	(Hnb)	alleles per locus	distinct MLH	diversity
**Total environment**	109	9	100	0.77 (0.16)	13.6	83	0.82
Precipitation	69	6	63	0.78 (0.14)	12.3	54	0.85
Snowpack	10	3	7	0.69 (0.18)	4	6	0.83
Fresh water	12	0	12	0.64 (0.29)	4.5	9	0.75
Epilithic biofilm	2	0	2	0.44 (0.33)	1.6	2	1
Litter	3	0	3	0.62 (0.26)	2.4	3	1
Plant debris	1	0	1	0	1.0	1	nd
Wild plants	7	0	7	0.57 (0.27)	3.3	7	1
Bird feather	5	0	5	0.38 (0.27)	2.1	5	1
**Agricultural strains**	327	6	321	0.76 (0.18)	15.6	186	0.58


**Table 3:** Allelic richness (AR) and unbiased gene diversity (Hnb) of environmental and agricultural strains of *B. cinerea*, at each of the nine microsatellite loci (corrected for the smallest sample size: 100).

	BC1		BC2		BC3		BC4		BC5		BC6		BC7		BC9		BC10	
									
	AR	Hnb	AR	Hnb	AR	Hnb	AR	Hnb	AR	Hnb	AR	Hnb	AR	Hnb	AR	Hnb	AR	Hnb
Environmental	21.0	0.91	15.0	0.87	11.0	0.84	11.0	0.84	12.0	0.87	25.0	0.83	11.0	0.81	10.0	0.55	14.0	0.86
Agricultural	21.2	0.87	15.8	0.89	15.2	0.84	15.2	0.84	12.4	0.81	21.4	0.84	11.0	0.85	9.6	0.47	16.8	0.86


**FIGURE 1:**
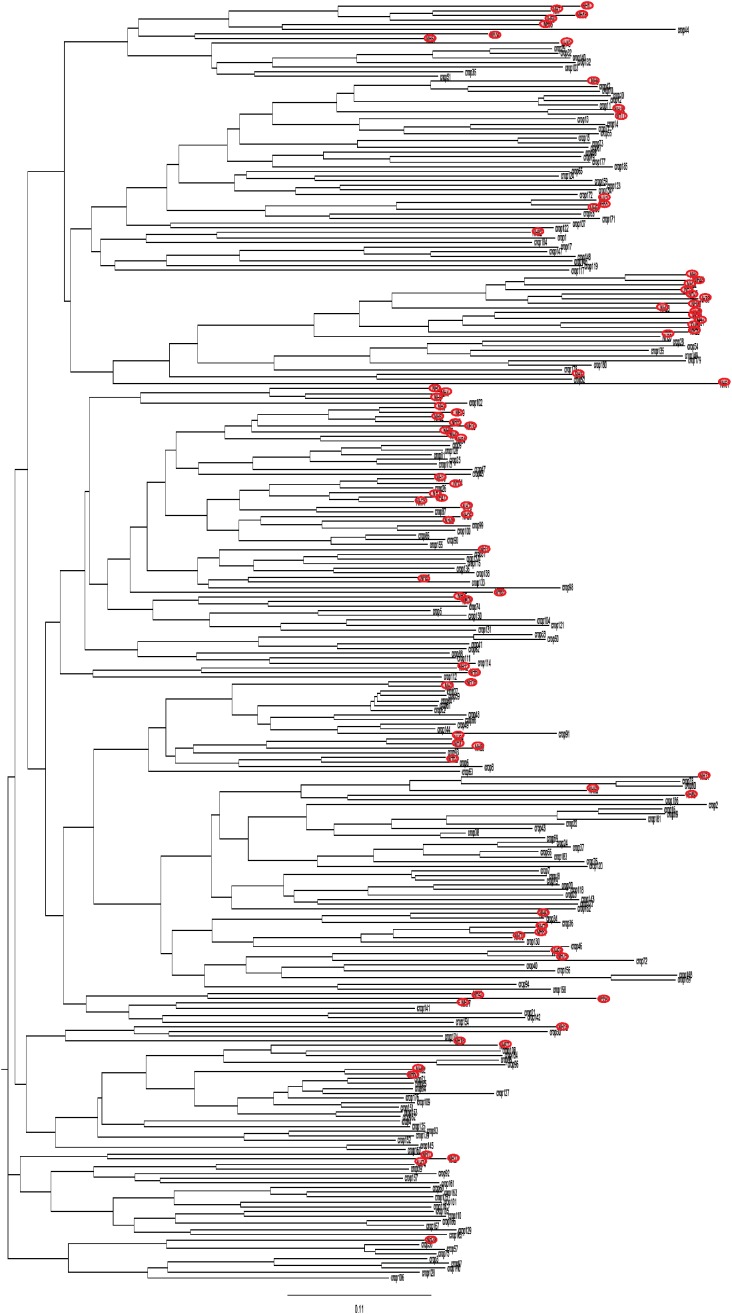
Neighbor-joining tree showing the genetic distance between strains from non-agricultural habitats (red circle) and from crops, based on microsatellite markers.

The distribution of the strains collected from crops and those collected from non-agricultural habitats in the NJ tree shows that they were widely intermixed in the tree regardless of their origin (Figure 1). This result is supported by the low level of genetic differentiation (*R*_ST_ = 0.021, *P* = 0.019) between these two groups of strains.

### Environmental Strains Represent a Greater Variability in Aggressiveness and an Overall Greater Mean Aggressiveness Than Agricultural Strains

**FIGURE 2:**
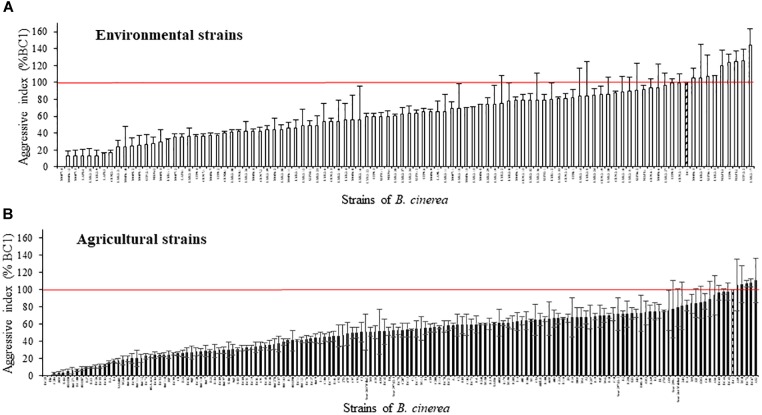
Aggressiveness of 128 environmental strains **(A)** and 156 agricultural strains **(B)** of *B. cinerea* on tomato plant in controlled condition (21°C, RH > 80%, photoperiod 14 h, 114 μmol m^-2^ s^-1^). Index of aggressiveness (in %) was calculated for each strain, relative to the reference strain BC1 (hatched). The horizontal red bar corresponds to an index of aggressiveness of 100% (value of BC1). Each value represents the mean of two or three independent repetitions. The vertical bar associated to each histogram correspond to SE.

Based on the lesion expansion on the stem of the tomato plants, different levels of aggressiveness were observed within the 128 environmental strains and the 156 agricultural strains of *B. cinerea* tested (Figure 2). For each of the population of strains (agricultural strains and environmental strains), significant differences were observed among strains (ANOVA, *P* < 0.0001). MOP7-4 isolated in rainfall in 2010, and H6 and BC25 isolated on diseased tomato plants in 1991 were not able to generate symptoms on the stem of the plant, suggesting that they are hypo-aggressive on tomato stems. However, these strains were able to infect the tomato petiole stubs (data not shown). The index of aggressiveness for the 284 strains of *B. cinerea* ranged from 0 to 144% on potted tomato plants with the wider range of aggressiveness for environmental strains compared to agricultural strains (Table 4). The mean disease severity caused by strains from environmental substrates was statistically identical to the severity of disease caused by strains from tomato, but was significantly greater than the severity caused by strains from grape or other crops. Globally, environmental strains are significantly more aggressive than agricultural strains on plants (58.9% vs. 50.4%, *P* = 0.009).

**Table 4:** Mean and range of aggressiveness of strains of *B. cinerea* collected on various substrates.

Substrate	Number of strains	Mean Ia^∗^ ± SE	Minimum	Maximum	Median
Total	284	54.2 ± 1.6	0.0	144.3	54.8
Environmental	128	58.9 ± 2.5^a^	0.0	144.3	58.2
Tomato	101	59.5 ± 2.4^a^	0.0	110.9	61.7
Grape	33	25.8 ± 2.5^c^	3.1	72.5	25.7
Other plants	22	45.7 ± 4.7^b^	11.8	97.6	43.4
ANOVA (*p*-value)		<0.0001			


*^∗^Ia (index of aggressiveness) is expressed in % relative to the reference strain BC1. Values with the same uppercase letter are not significantly different according to Newman-Keuls multiple range test (*P* < 0.05).*

### Environmental Strains Grow Faster and Sporulate Less Abundantly but Do Not Differ in Sclerotial Production Compared to Agricultural Strains

**FIGURE 3:**
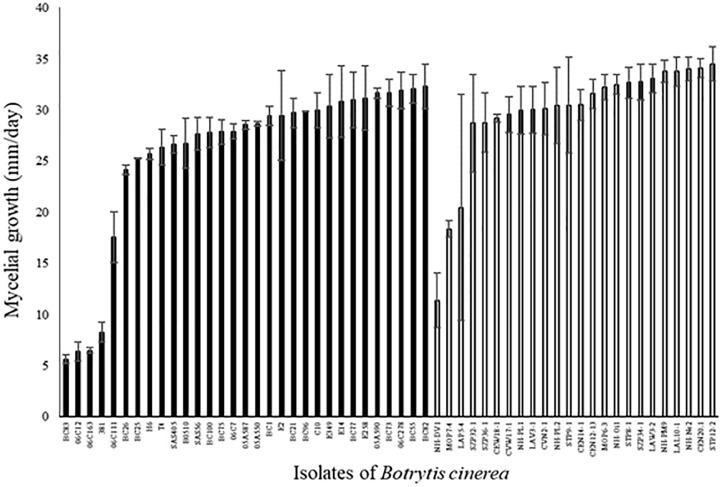
Mycelial growth (mm/day) of 31 agricultural (black) and 24 environmental (white) strains of *B. cinerea* on PDA medium in controlled condition (21°C, photoperiod 14 h, 114 μmol m^-2^ s^-1^). Each value represents the mean of two independent repetitions. The vertical bar associated to each histogram correspond to SE.

Among the 31 agricultural and 24 environmental strains tested, the mycelial growth between the first and the third day after the plating of mycelial plug varied widely among strains (Figure 3; ANOVA, *P* < 0.0001 for each set of strains). Four strains (BC83, 06C12, 06C163, and S381) had a mycelial growth rate significantly lower than the other strains. These four strains were all isolated from diseased plants (rose, grape and tomato). Globally, mycelial growth rate was significantly higher for environmental strains compared to agricultural strains (29.8 ± 0.4 vs. 25.7 ± 0.4; *P* < 0.0001). A significant correlation was detected between aggressiveness of strains on tomato plants and their mycelial growth on PDA medium (*r*^2^ = 0.12, *P* = 0.009 for all the strains). However, this correlation is not significant for each set of strains taken independently (*r*^2^ = 0.09, *P* = 0.17 for environmental strains and *r*^2^ = 0.11, *P* = 0.08 for agricultural strains).

**FIGURE 4:**
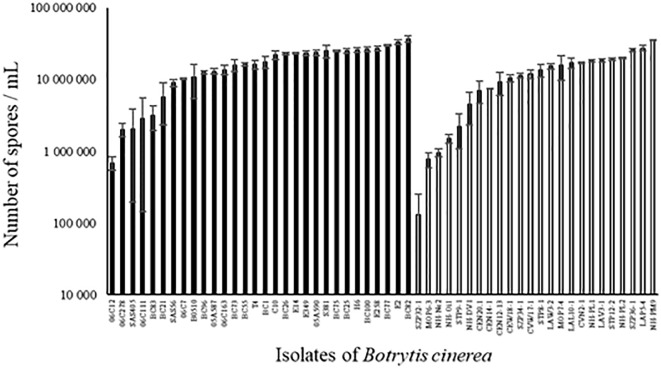
Number of spores produced on PDA medium after 14 days of incubation in controlled condition (21°C, photoperiod 14 h, 114 μmol m^-2^ s^-1^) by 30 agricultural (black) and 24 environmental (white) strains of *B. cinerea*. Each value represents the mean of three replicates. The vertical bar associated to each histogram correspond to SE.

All strains of *B. cinerea* tested were able to sporulate on PDA medium but the number of spores produced varied widely among strains, both for environmental and for agricultural strains (Figure 4; ANOVA, *P* < 0.0001 for each population of strains). Globally, sporulation was significantly lower for environmental strains compared to agricultural strains (12.9 ± 1.1 vs. 17.4 ± 1.1 million spores/mL; *P* = 0.005). There was no significant correlation between aggressiveness of strains on tomato plants and sporulation (*r*^2^ = 0.00001, *P* = 0.98 for all strains).

**FIGURE 5:**
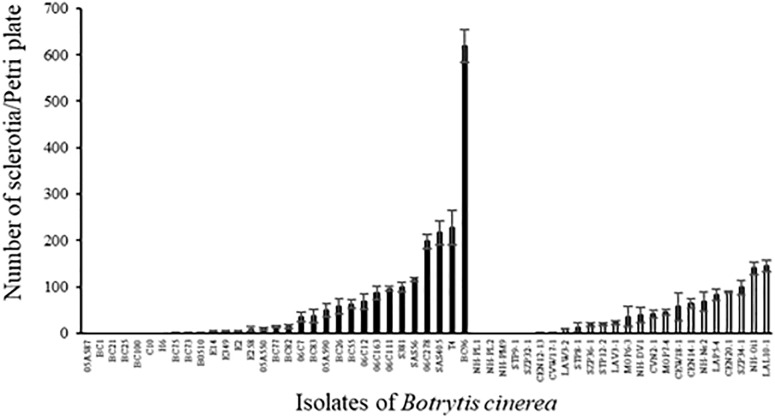
Number of sclerotia produced by 31 agricultural (black) and 24 environmental (white) strains of *B. cinerea* on PDA medium after 5 weeks of incubation in controlled condition at 21°C (1 week with a photoperiod of 14 h at 114 μmol m^-2^ s^-1^ and 4 weeks in the dark) and 5 weeks of incubation at 4°C in the dark. Each value represents the mean of three replicates. The vertical bar associated to each histogram correspond to SE.

The number of sclerotia produced on PDA medium varied greatly among strains (Figure 5; ANOVA, *P* < 0.0001 for both environmental and agricultural strains) and the average number of sclerotia produced was not different between the two sets of strains (41.1 ± 5.5 and 65.5 ± 12.6 for environmental and agricultural strains, respectively, *P* = 0.11). Twelve strains of *B. cinerea* (7 from diseased plants and 5 from non-agricultural context) were not able to produce sclerotia under the conditions used here and one strain (BC96 isolated from hydrangea) produced a significantly higher number of sclerotia than all the other strains. We found a significant negative relationship for all strains between the aggressiveness and the number of sclerotia produced (*r*^2^ = 0.09, *P* = 0.031). This correlation is highly significant for the 24 non-agricultural strains (*r*^2^ = 0.47, *P* = 0.0002) while it was not significant for the 31 agricultural strains (*r*^2^ = 0.03, *P* = 0.33), suggesting that the relationship between aggressiveness and sclerotial production differ among these two populations of strains.

## Discussion

This study is the first systematic census of the fungal plant pathogen *B. cinerea* in diverse non-agricultural environments that could come into contact with agriculture such as snowpack, fresh water from river and lake, epilithic biofilms in mountain streams, leaf litter, plant debris and bird feathers. These results complement the prior detection of *B. cinerea* in the diverse substrates described in the introduction. It also contributes to information about sources of *B. cinerea* in precipitation (Monteil et al., 2014) and corroborates reports about its capacity to colonize and survive internally in plants without causing any symptoms (Van Kan et al., 2014), traits that are essential for an endophytic phase. Our results illustrate that *B. cinerea* can survive in environmental reservoirs outside of agriculture *sensu stricto* and possibly in the absence of susceptible plants. This knowledge contributes to the growing notion that the life history of plant pathogens should be viewed from a wider angle than their direct interaction with plants in contexts that are favorable for disease. Furthermore, the relative ease with which we found strains of *B. cinerea* in environmental reservoirs highlights how defining this fungus as a plant pathogen skews research questions on its life history and ecology in spite of the numerous previous reports of its presence in a wide range of diverse habitats.

Our results also suggest that the environmental reservoirs are probable sources of inoculum. Previous reports of the genetic diversity of *B. cinerea* from wild plants (e.g., primrose, bramble, dandelion, *Hypochaeris radicata*, *Plantago lanceolata*, and *Sonchus asper*), illustrated the potential reservoirs of inoculum for crops on wild plants and weeds in agricultural systems (Fournier and Giraud, 2008; Rajaguru and Shaw, 2010; Walker et al., 2015; Wessels et al., 2016), but there have been no studies on non-agricultural sources. Phylogenetic comparisons of strains from crop plants and from environmental substrates presented in this study revealed that strains from crops and from the environment could not be distinguished, thereby supporting the hypothesis that strains from these different sources mix. Furthermore, the genetic diversity of strains outside of agriculture was just as broad as within agriculture (Table 5; Bardin et al., 2014), suggesting that there are no strong selective pressures that remarkably distinguish these two categories of habitats. These results are consistent with those previously published for *B. cinerea* in different agricultural contexts, using the same genotyping tools developed by Fournier et al. (2002). In such studies, the haplotypic diversity (ratio of the number of different haplotypes over the total number of characterized strains) varied from 0.37 to 1.00. In the present study, this index was high, with a value of 0.82, comparable to what is observed in populations of *B. cinerea* collected on diseased plant in open field situations. It reflects the scarcity of strains with identical genotypic profiles in the non-agricultural context. Although we have demonstrated that strains from a non-agricultural context cannot be genetically distinguished from strains from diseased plants, various questions remain about the rate of flux of strains between non-agricultural environment and crops and on the contribution of environmental reservoirs to epidemics.

**Table 5:** Genetic diversity reported for strains of *B. cinerea* collected from different origins and characterized using the same microsatellite markers developed by Fournier et al. (2002), as in this study.

		Number of	Gene	Mean number of	Haplotypic
Strain origin^a^	Localization	isolates	diversity	alleles per locus	diversity^b^	Reference
**Environmental**	France	100	0,77	13.6	0,82	Present study
Pear orchard (OF)	South-Africa	181	0.69	5.49–7.76	0.50	Wessels et al., 2016
Grapevine (OF)	Italy	317	na^c^	na	0.92	Campia et al., 2017
Grapevine (OF)	China	135	0.29	na	0.94	Zhang et al., 2018
Tomato, lettuce (G)	France	86	0.62–0.77	4.70–9.20	0.37–0.69	Leyronas et al., 2015b
Tomato (G)	France	170	0.76–0.77	11.6–11.3	0.55–0.65	Leyronas et al., 2015a
Tomato (G)	Algeria	170	0.57	8.22	0.63	Adjebli et al., 2015
Soil (G)	France	66	0.68	8.5	0.58	Leyronas et al., 2015c
Air (OF)	France	616	0.71	19.11	0.72	Leyronas et al., 2015d


*The References presented here range from 2015 to 2018. For information on previous references, see Bardin et al. (2014). ^*a*^Indication between brackets indicates the type of crop. OF, open field; G, greenhouse. ^*b*^Haplotypic diversity index directly available or computed (on the basis of reported information) as the ratio of (number of distinct MLG – 1) over (sample size – 1). ^*c*^na, data not available.*

In tests to determine the severity of disease that strains could induce on tomato stems, the mean disease severity caused by strains from environmental substrates was statistically greater than the severity caused by strains from diseased crops (*P* = 0.009). It suggests that there are no selective pressures for strains of *B. cinerea* to lose pathogenicity while they are in contexts where they do not cause plant disease or are not even colonizing plants. On the other hand, the environment seems to select for more rapid mycelial growth whereas cropping contexts select for more abundant sporulation. This suggests that there are fitness or survival benefits for these traits in each of the respective environments. This hypothesis is reinforced by the fact that mycelial growth is positively correlated with aggressiveness. Therefore, it suggests that the non-agricultural environment could select strains for factors that would facilitate their pathogenicity on plants. It would be interesting to determine how much time is needed for mycelial growth and sporulation rates to change under the selection pressures of each environment and if in fact these traits are fixed or due to epigenetic processes. This could give clues to the amount of time that strains from these habitats have been localized in one habitat or another. Interestingly, a trade-off between the aggressiveness and the number of sclerotia produced was observed for the environmental strains of *B. cinerea*. This suggests that the least aggressive environmental strains compensate this low level of aggressiveness by their high ability to survive in the environment in the absence of host plants.

Our results are consistent with those concerning the life history of the bacterial plant pathogen *P. syringae* (Morris et al., 2013) in that highly diverse populations of strains that maintain their pathogenic potential can persist outside of agriculture in association with substrates other than plants. These findings set the stage for new perspectives on factors that foster to the evolution and emergence of pathogenic potential in microorganisms, on disease epidemiology and on the factors that contribute to the durability of disease control methods. They also beg for the development of systematic approaches to widen our understanding of the life history of plant pathogens in general beyond the context of agriculture.

## Author Contributions

MB, CT, and CM achieved the sampling of environmental strains. MB and CT realized the phenotypic experiments and the associated data analysis. CL carried out microsatellite genotyping and the genetic data analysis. MB coordinated the writing of the manuscript. MB, CL, and CM wrote the manuscript. All authors reviewed the manuscript.

## Conflict of Interest Statement

The authors declare that the research was conducted in the absence of any commercial or financial relationships that could be construed as a potential conflict of interest.

## References

[B1] Adjebli A. Leyronas C. Aissat K. Nicot P. C. (2015). Comparison of *Botrytis cinerea* populations collected from tomato greenhouses in northern algeria. *J. Phytopathol.* 163 124–132. 10.1111/jph.12289

[B2] Ali-Shtayeh M. S. Salameh A. A. M. Abu-Ghdeib S. I. Jamous R. M. (2001). Hair and scalp mycobiota in school children in Nablus area. *Mycopathologia* 150 127–135. 10.1023/a:1010989431375 11469760

[B3] Amato P. Parazols M. Sancelme M. Laj P. Mailhot G. Delort A. M. (2007). Microorganisms isolated from the water phase of tropospheric clouds at the Puy de Dome: major groups and growth abilities at low temperatures. *FEMS Microbiol. Ecol.* 59 242–254. 10.1111/j.1574-6941.2006.00199.x 17328765

[B4] Arnaud-Haond S. Belkhir K. (2007). Genclone: a computer program to analyse genotypic data, test for clonality and describe spatial clonal organization. *Mol. Ecol. Notes* 7 15–17. 10.1111/j.1471-8286.2006.01522.x

[B5] Arnaud-Haond S. Duarte C. M. Alberto F. Serrao E. A. (2007). Standardizing methods to address clonality in population studies. *Mol. Ecol.* 16 5115–5139. 10.1111/j.1365-294X.2007.03535.x 17944846

[B6] Azmi O. R. Seppelt R. D. (1998). The broad-scale distribution of microfungi in the windmill islands region, continental antarctica. *Polar Biol.* 19 92–100. 10.1007/s003000050219

[B7] Bardin M. Decognet V. Nicot P. C. (2014). Remarkable predominance of a small number of genotypes in greenhouse populations of *Botrytis cinerea*. *Phytopathology* 104 859–864. 10.1094/PHYTO-10-13-0271-R 24521484

[B8] Barnett H. L. (1998). *Illustrated Genera of Imperfect Fungi*, 4th Edn Saint Paul, MN: American Phytopathological Society.

[B9] Bartlett K. H. Kennedy S. M. Brauer M. Van Netten C. Dill B. (2004). Evaluation and a predictive model of airborne fungal concentrations in school classrooms. *Ann. Occup. Hyg.* 48 547–554. 10.1093/annhyg/meh051 15302620

[B10] Belirkh K. Borsa P. Chikhi L. Raufaste N. Bonhomme F. (1996–2004). *Genetix 4.05 Logiciel Sous Windows Tm Pour La Génétique Des Populations*. Montpellier: Université de Montpellier.

[B11] Campia P. Venturini G. Moreno-Sanz P. Casati P. L. T. S. (2017). Genetic structure and fungicide sensitivity of *Botrytis cinerea* populations isolated from grapevine in northern Italy. *Plant Pathol.* 66 890–899. 10.1111/ppa.12643

[B12] Cavalli-Sforza L. L. Edwards A. W. F. (1967). Phylogenetic analysis: models and estimation procedures. *Am. J. Hum. Genet.* 19 233–257. 6026583 PMC1706274

[B13] Decognet V. Bardin M. Trottin-Caudal Y. Nicot P. C. (2009). Rapid change in the genetic diversity of *Botrytis cinerea* populations after the introduction of strains in a tomato glasshouse. *Phytopathology* 99 185–193. 10.1094/PHYTO-99-2-0185 19159311

[B14] Edel-Hermann V. Sautour M. Gautheron N. Laurent J. Aho S. Bonnin A. (2016). A clonal lineage of *Fusarium oxysporum* circulates in the tap water of different French hospitals. *Appl. Environ. Microbiol.* 82 6483–6489. 10.1128/AEM.01939-16 27663024 PMC5066365

[B15] Edwards S. G. Seddon B. (2001). Selective media for the specific isolation and enumeration of *Botrytis cinerea* conidia. *Lett. Appl. Microbiol.* 32 63–66. 10.1046/j.1472-765x.2001.00857.x 11169044

[B16] Elad Y. Pertot I. Cotes Prado A. M. Stewart A. (2016). “Plant hosts of *Botrytis* spp,” in *Botrytis - The Fungus, the Pathogen Ans Its Management in Agricultural Systems*, eds Fillinger S. Elad Y. (Berlin: Springer), 413–486. 10.1007/978-3-319-23371-0_20

[B17] Excoffier L. Laval G. Schneider S. (2005). Arlequin ver. 3.0: an integrated software package for population genetics data analysis. *Evol. Bioinform.* 1 47–50. 10.1177/117693430500100003 19325852 PMC2658868

[B18] Fermaud M. Lemenn R. (1989). Association of *Botrytis cinerea* with grape berry moth larvae. *Phytopathology* 79 651–656. 10.1094/Phyto-79-651

[B19] Fournier E. Giraud T. (2008). Sympatric genetic differentiation of a generalist pathogenic fungus, *Botrytis cinerea*, on two different host plants, grapevine and bramble. *J. Evol. Biol.* 21 122–132. 10.1111/j.1420-9101.2007.01462.x 18028352

[B20] Fournier E. Giraud T. Loiseau A. Vautrin D. Estoup A. Solignac M. (2002). Characterization of nine polymorphic microsatellite loci in the fungus *Botrytis cinerea* (Ascomycota). *Mol. Ecol. Notes* 2 253–255. 10.1046/j.1471-8286.2002.00207.x

[B21] Gaylarde C. C. Bento F. M. Kelley J. (1999). Microbial contamination of stored hydrocarbon fuels and its control. *Rev. Microbiol.* 30 1–10. 10.1590/s0001-37141999000100001

[B22] Gaylarde C. C. Morton L. H. G. (1999). Deteriogenic biofilms on buildings and their control: a review. *Biofouling* 14 59–74. 10.1080/08927019909378397

[B23] Goudet J. (1995). FSTAT (Vers.1.2): a computer program to calculate F-statistics. *J. Heredity* 86 485–486. 10.1093/oxfordjournals.jhered.a111627

[B24] Hansen E. M. Reeser P. W. Sutton W. (2012). *Phytophthora* beyond agriculture. *Annu. Rev. Phytopathol.* 50 359–378. 10.1146/annurev-phyto-081211-172946 22681450

[B25] Leyronas C. Bardin M. Duffaud M. Nicot P. C. (2015a). Compared dynamics of grey mould incidence and genetic characteristics of *Botrytis cinerea* in neighbouring vegetable greenhouses. *J. Plant Pathol.* 97 439–447.

[B26] Leyronas C. Bryone F. Duffaud M. Troulet C. Nicot P. C. (2015b). Assessing host specialization of *Botrytis cinerea* on lettuce and tomato by genotypic and phenotypic characterization. *Plant Pathol.* 64 119–127. 10.1111/ppa.12234

[B27] Leyronas C. Duffaud M. Pares L. Jeannequin B. Nicot P. C. (2015c). Flow of *Botrytis cinerea* inoculum between lettuce crop and soil. *Plant Pathol.* 64 701–708. 10.1111/ppa.12284

[B28] Leyronas C. Halkett F. Nicot P. C. (2015d). Relationship between the genetic characteristics of *Botrytis* sp. airborne inoculum and meteorological parameters, seasons and the origin of air masses. *Aerobiologia* 31 367–380. 10.1007/s10453-015-9370-x

[B29] Leyronas C. Duffaud M. Nicot P. C. (2012). Compared efficiency of the isolation methods for *Botrytis cinerea*. *Mycol. Int. J. Fungal Biol.* 3 221–225.

[B30] Leyronas C. Nicot P. C. (2013). Monitoring viable airborne inoculum of *Botrytis cinerea* in the South-East of France over 3 years: relation with climatic parameters and the origin of air masses. *Aerobiologia* 29 291–299. 10.1007/s10453-012-9280-0

[B31] Louis C. Girard M. Kuhl G. LopezFerber M. (1996). Persistence of *Botrytis cinerea* in its vector *Drosophila melanogaster*. *Phytopathology* 86 934–939. 10.1094/Phyto-86-934

[B32] Lugauskas A. Krikstaponis A. (2004). Microscopic fungi found in the libraries of vilnius and factors affecting their development. *Indoor Built Environ.* 13 169–182. 10.1177/1420326x04045274

[B33] Monteil C. L. Bardin M. Morris C. E. (2014). Features of air masses associated with the deposition of *Pseudomonas syringae* and *Botrytis cinerea* by rain and snowfall. *ISME J.* 8 2290–2304. 10.1038/ismej.2014.55 24722630 PMC4992071

[B34] Monteil C. L. Yahara K. Studholme D. J. Mageiros L. Méric G. Swingle B. (2016). Population-genomic insights into emergence, crop-adaptation, and dissemination of *Pseudomonas syringae* pathogens. *Microb. Genom.* 2:e000089. 10.1099/mgen.0.000089 28348830 PMC5359406

[B35] Morris C. E. Bardin M. Kinkel L. L. Moury B. Nicot P. C. Sands D. C. (2009). Expanding the paradigms of plant pathogen life history and evolution of parasitic fitness beyond agricultural boundaries. *PLoS Pathog.* 5:e1000693. 10.1371/journal.ppat.1000693 20041212 PMC2790610

[B36] Morris C. E. Monteil C. L. Berge O. (2013). The life history of *Pseudomonas syringae*: linking agriculture to Earth system processes. *Annu. Rev. Phytopathol.* 51 85–104. 10.1146/annurev-phyto-082712-102402 23663005

[B37] Page R. D. M. (1996). Treeview: an application to display phylogenetic trees on personal computers. *Comp. Appl. Biosci.* 12 357–358. 8902363 10.1093/bioinformatics/12.4.357

[B38] Rajaguru B. A. P. Shaw M. W. (2010). Genetic differentiation between hosts and locations in populations of latent *Botrytis cinerea* in southern England. *Plant Pathol.* 59 1081–1090. 10.1111/j.1365-3059.2010.02346.x

[B39] Shchipanov N. A. Aleksandrov D. Y. Aleksandrova A. V. (2006). Dispersal of micromycete spores by small mammals. *Zool. Zhurnal.* 85 101–113.

[B40] Slatkin M. (1995). A measure of population subdivision based on microsatellite allele frequencies. *Genetics* 139 1463–1463. 7705646 10.1093/genetics/139.1.457 PMC1206343

[B41] Van Kan J. A. L. Shaw M. W. Grant-Downton R. T. (2014). *Botrytis* species: relentless necrotrophic thugs or endophytes gone rogue? *Mol. Plant Pathol.* 15 957–961. 10.1111/mpp.12148 24754470 PMC6638755

[B42] Walker A. S. Gautier A. Confais J. Martinho D. Viaud M. Le Pêcheur P. (2011). *Botrytis pseudocinerea*, a new cryptic species causing gray mold in French vineyards in sympatry with *Botrytis cinerea*. *Phytopathology* 101 1433–1445. 10.1094/PHYTO-04-11-0104 21830954

[B43] Walker A. S. Gladieux P. Decognet V. Fermaud M. Confais J. Roudet J. (2015). Population structure and temporal maintenance of the multihost fungal pathogen *Botrytis cinerea*: causes and implications for disease management. *Environ. Microbiol.* 17 1261–1274. 10.1111/1462-2920.12563 25040694

[B44] Wessels B. A. Linde C. C. Fourie P. H. Mostert L. (2016). Genetic population structure and fungicide resistance of *Botrytis cinerea* in pear orchards in the Western Cape of South Africa. *Plant Pathol.* 65 1473–1483. 10.1111/ppa.12523

[B45] Williamson B. Tudzynski B. Tudzynski P. van Kan J. A. L. (2007). *Botrytis cinerea*: the cause of grey mould disease. *Mol. Plant Pathol.* 8 561–580. 10.1111/j.1364-3703.2007.00417.x 20507522

[B46] Zhang Y. Li X. Shen F. Xu H. Li Y. Liu D. (2018). Characterization of *Botrytis cinerea* isolates from grape vineyards in China. *Plant Dis.* 102 40–48. 10.1094/PDIS-01-17-0062-RE 30673451

